# Use of Geographic Information Systems to Explore Associations between Neighborhood Attributes and Mental Health Outcomes in Adults: A Systematic Review

**DOI:** 10.3390/ijerph18168597

**Published:** 2021-08-14

**Authors:** Young-Shin Park, Barbara J. McMorris, Lisiane Pruinelli, Ying Song, Merrie J. Kaas, Jean F. Wyman

**Affiliations:** 1 School of Nursing, University of Minnesota, Minneapolis, MN 55455, USA; mcmo0023@umn.edu (B.J.M.); pruin001@umn.edu (L.P.); kaasx002@umn.edu (M.J.K.); wyman002@umn.edu (J.F.W.); 2 Mo-Im Kim Nursing Research Institute, College of Nursing, Yonsei University, Seoul 03722, Korea; 3 Geography, Environment and Society, University of Minnesota, Minneapolis, MN 03722, USA; yingsong@umn.edu

**Keywords:** neighborhood attributes, depressive symptoms, psychological distress, geographic information systems

## Abstract

Background: Neighborhood attributes are increasingly recognized as factors shaping mental health in adults. Geographic information systems (GIS) offer an innovative approach for quantifying neighborhood attributes and studying their influence on mental health outcomes. Our aim was to describe GIS applications used in neighborhood-related mental health research and how neighborhood attributes are related to depressive symptoms or psychological distress in community-residing adults. Methods: We conducted a systematic review of studies published in English that included GIS techniques and a validated questionnaire of depressive symptoms or psychological distress. Medline, PsycInfo, Embase, Scopus, CINAHL, GEOBASE, and Compedex were searched to June 2020. Study quality was assessed by a modification of the Joanna Briggs Institute’s Checklist for Analytical Cross-sectional Studies. Results: Thirty-two studies met the inclusion criteria. Studies varied in definitions of neighborhood and GIS-derived measurements of neighborhood attributes. Neighborhood attributes were significantly associated with mental health outcomes, although findings were not consistent. Moderating factors (e.g., gender, living conditions) significantly influenced depressive symptoms or psychological distress. Conclusion: Neighborhood attributes are important factors influencing mental health in adults. Consensus may be needed on how to standardize the neighborhood unit or GIS-derived measures of neighborhoods in order to explain depression or psychological distress in diverse adult populations.

## 1. Introduction

Globally, depression is the most common mental health disorder. In the United States, prevalence estimates from 2013 to 2016 indicate that approximately 8% of community-residing adults reported current depression [1], with a lifetime prevalence of depression of 20% [2]. Depression complicates chronic conditions such as cardiovascular diseases, stroke, respiratory diseases, auto-immune diseases, and cognitive impairment [3,4,5]. It is one of the leading causes of disability or suicide [6]. The economic burden of depression reached USD 210.5 billion in the US and it continues to increase the total burden of diseases [6,7].

Depression is influenced by an interaction of biological, psychological, and social factors [8]. While the impact of individual factors for depression is becoming clear, social influences such as neighborhood characteristics on depression are not well-known. Because depression is a stress-related disorder, the psychological consequences of living in a particular neighborhood may differ across regions [8,9]. Recently, neighborhood factors such as unemployment, an unhealthy food environment, insecure housing, or an unsafe environment have gained attention for their influence on depression in adults [9,10]. Identifying the neighborhood risks or protective factors that are associated with depression and other mental health symptoms is important for designing population-based interventions that can be used by health and social policy makers [11].

From an environmental perspective, accumulated and long-term exposure to a neighborhood with more stressors such as lack of safety, physical hazards, poverty, or low levels of social support may be related to an increase in depression [12]. Specific neighborhood attributes may play a role either as a supportive or a detrimental factor influencing depression [13]. For instance, living in a walkable neighborhood may promote physical activity, which in turn decreases the risk of depression [14,15]. On the contrary, living in disadvantaged neighborhoods such as poor, disorganized, or violent neighborhoods and neighborhoods with fewer services may increase the risk of depression [16].

Four prior reviews examined neighborhood effects on mental health outcomes in adults [17,18] or older adults [19,20]. Neighborhood attributes associated with depressive symptoms included socioeconomic composition, neighborhood context (e.g., collective efficacy, residential stability, crime, safety), and built environment (e.g., housing, walkability, or land-use mix) [17,18,20]. These reviews had methodological limitations including the use of limited search strategies and the lack of an objective measure of neighborhood attributes. Since these reviews were published, an increased number of studies have used Geographical Information Systems (GIS) to quantify neighborhood attributes and examine their effects on depressive symptoms and psychological distress. GIS, an innovative technology, relies on computer-assisted systems for mapping, visualizing, integrating, and analyzing geographic data to store or compute and display spatial relationships between attributes; analyze spatial data; and integrate spatial data from different sources [21]. It offers the advantage of understanding spatial characteristics and geographical relationships among neighborhood attributes or incorporating neighborhood contexts to explain health outcomes.

Previous studies on the relationships between neighborhood characteristics and mental health outcomes were primarily based on empirical evidence to support the research questions [22,23,24,25,26], and are limited by the lack of a theoretical or conceptual framework guiding the study design and methodology. Researchers have characterized neighborhood attributes in various ways [20]. Galster [27] identified 10 comprehensive categories of neighborhood attributes, which provide a useful approach in guiding the analyses of neighborhood attributes measured using GIS.

Therefore, the purpose of this literature review was to update the evidence on how neighborhood attributes affect depressive symptoms and psychological distress in adults, with a focus on using GIS in the spatial representation of neighborhoods and measurement of their attributes. This review has four aims: (1) describe GIS methods used to measure neighborhood attributes; (2) summarize operational definitions used for neighborhoods and the resulting geographical unit; and (3) examine how the neighborhood attributes measured by GIS are related to depressive symptoms and psychological distress in adults. Recommendations on how to measure neighborhood attributes using GIS and geographic units in future studies on neighborhood environments and mental health outcomes will be offered.

## 2. Methods

### 2.1. Overview

This systematic review examined studies using applications of GIS to investigate the relationships of neighborhood attributes to mental health outcomes in community-dwelling adults. For the first aim, neighborhood attributes were identified and organized using Galster’s [27] neighborhood attribute categories. For the second aim, the descriptions of GIS-derived measurements of neighborhood attributes and operational definitions of neighborhood were organized into a table along with attributes found from the previous step. To fulfill the last aim, common GIS techniques used, geographical unit and type were analyzed for their similarities or differences and for their relationships with mental health outcomes.

### 2.2. Protocol and Registration

This systematic review conforms to the Preferred Reporting Items for Systemic Reviews and Meta-analyses (PRISMA) Guidelines [28], with the review protocol registered in PROSPERO (CRD42020138798).

### 2.3. Search Strategy

**Figure 1:**
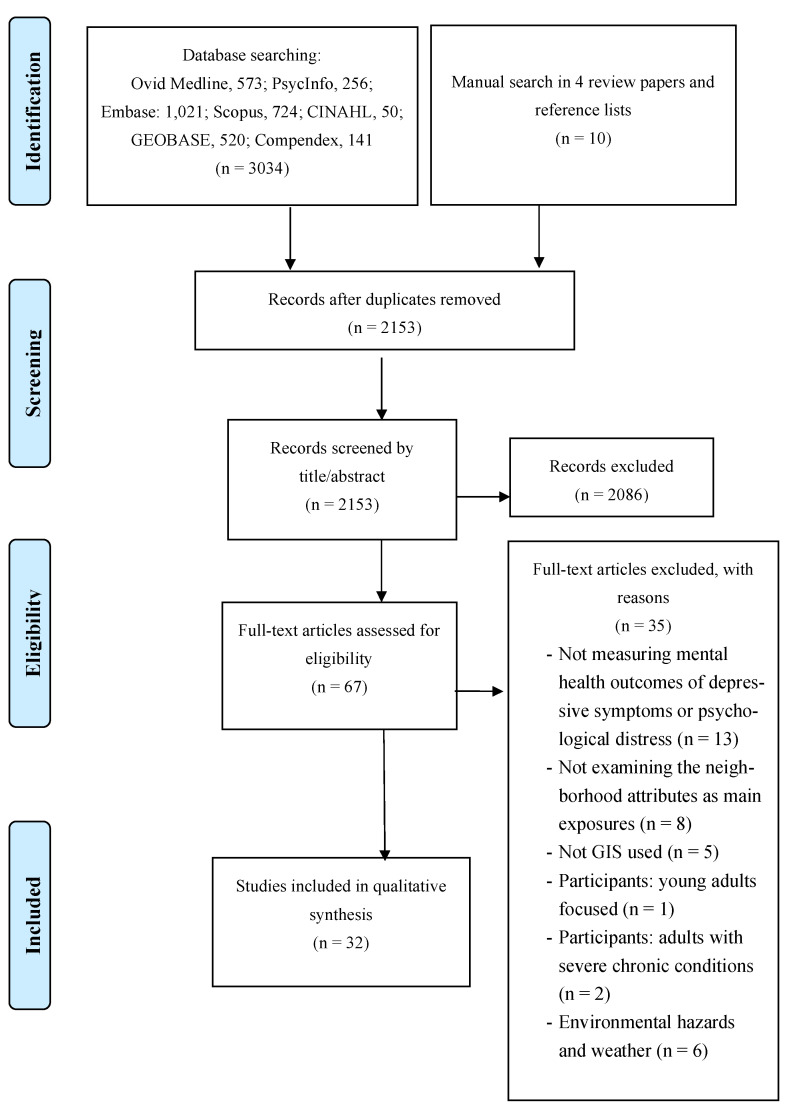
Flow chart on study selection process.

The search strategy was developed in consultation with a health science librarian. Seven databases in health and health-related research (Ovid Medline, PsycInfo, Embase, Scopus, CINAHL, GEOBASE, and Compedex) were searched up to June 2020 with no limitation of the year of publication (Figure 1). Keywords related to mental health outcomes included “mental health”, “mental disorders”, “mental health services”, “hospitals”, “psychiatric”, “depress*”, or “distress”. Additionally, keywords related to GIS included: “Geographic information systems”, “Geographic information system”, “Geographical information systems”, “Geographical information system”, “Geospatial”, “GIS”, or “spatial analysis” (Appendix A). Additionally, reference lists of included articles for review and review papers were searched manually [17,18,19,20].

### 2.4. Eligibility Criteria

Articles were included if they: (a) targeted mostly community-dwelling adults; (b) used a validated mental health outcome measure to assess depressive symptoms or psychological distress; (c) used GIS techniques in any study design phase; (d) included neighborhood attributes as main exposure; (e) included quantitative analysis; and (f) were peer-reviewed articles published in English. Articles were excluded if they focused on: (a) children or adolescents only; (b) pregnant women due to their temporary and unique risk factors associated with childbearing [29]; or (c) qualitative research, literature reviews, discussion papers or editorials.

### 2.5. Study Selection

Articles were selected using a two-step screening process. Titles and abstracts were assessed initially for eligibility. After removing duplicates and records meeting exclusion criteria, the remaining full-text articles were screened by two independent reviewers. Disagreements were resolved by discussion, with reasons for exclusion recorded.

### 2.6. Data Extraction

Data from eligible articles were extracted by one reviewer using a pre-defined and specifically developed spreadsheet for this review. A second reviewer checked data extracted in a random sample of 25% of articles. Extracted data included study details (author, year, country, study region, research questions/aims, study design); sample; data sources (population/neighborhood); mental health outcomes (measurement); neighborhood attributes; individual attributes (covariate/confounder); and GIS techniques, geographical unit, analytics, and results.

### 2.7. Quality Assessment

Quality of studies was appraised using a modified Checklist for Analytical Cross-sectional Studies developed by the Joanna Briggs Institute [30] (Table 1). Seven of the eight criteria were used; a criterion related to objective measurement of the condition was excluded from the Checklist because this item was not relevant to studies included for this review. Items were scored as “Yes,” “No,” “Unclear,” or “Not Applicable” by two independent reviewers, with disagreements resolved through discussion. Studies were deemed as having high methodologic quality when 80–100% of criteria were met, and moderate quality when 50–79% of criteria were met. No articles were excluded based on methodological rigidity.

### 2.8. Evidence Synthesis

A qualitative synthesis was conducted to analyze the findings of the included studies. Several articles based on the same study population but using different analytics [25,26,31,32,33,34] or different subsamples with varying neighborhood attributes [35,36,37,38,39] were considered as individual studies in the analyses.

Neighborhood attributes were identified and organized using Galster’s [27] neighborhood attribute categories: (1) residential/non-residential (structural characteristics of the residential and non-residential buildings), (2) infrastructure (characteristics of roads, sidewalks, etc.), (3) demographic (characteristics of populations such as age, racial/ethnic, etc.), (4) socioeconomic (class status characteristics such as income, occupation, or education), (5) public services ( services such as schools, administration, parks, and recreation, etc.), (6) environmental (topological characteristics, degree of land, air, water, and noise pollution, etc.), (7) proximity (access to major destinations of work, shopping, etc.), (8) political (extent local political networks are influenced), (9) social-interactive (degree or quality of interpersonal networks or social participation), and (10) emotional (characteristics such as sense of residence identification with significance). Descriptions of GIS-derived measurements of neighborhood attributes and operational definitions of the neighborhood were organized along with attributes found from the previous step. Common GIS techniques used and geographical unit and type were analyzed for their similarities or differences and their relationships with mental health outcomes. A significance level for distinguishing the main and the moderating effects of neighborhood attributes on mental health outcomes was set at the level of 0.05 to select significant neighborhood attributes related to depressive symptoms or psychological distress.

## 3. Results

### 3.1. Study Identification

A total of 3739 articles were located from databases and 10 additional articles identified from the manual search. After removing duplicates, 2153 articles were screened. Most articles were excluded through title and abstract screening, with 67 articles included in the full-text review. Finally, 30 articles were determined eligible to be included in the review (Figure 1). 

### 3.2. Study Characteristics

The 32 included studies were conducted in 12 countries: Australia (n = 8); Canada (n = 2); China (n = 3); Finland (n = 1); France (n = 1); the Netherlands (n = 1); New Zealand (n = 1); South Africa (n = 2); Spain (n = 1); Sweden (n = 2); the United Kingdom (n = 2); and the United States (n = 8) (Table 2). Two studies excluded urban areas; 30 studies covered areas from urban to rural areas. The majority of studies (63%) did not include a theoretical or conceptual framework. Over one-third of the studies (n = 12) used a psycho-evolutionary theory (n = 1); social-ecological framework (n = 5); social stress model (n = 1); attention restoration/stress reduction theory (n = 1); social disorganization theory (n = 1); behavioral model of health services use (n = 1); stress process in neighborhood context (n = 1); and one conceptual framework (n = 1). Approximately 70% of studies (n = 22) adopted a cross-sectional design, with nine studies (30%) using a longitudinal cohort design.

Eighteen studies targeted adults mostly aged 18+ years, six studies targeted adults middle-aged and older, and eight studies targeted older adults. Most studies (n = 28) included participants regardless of gender or race/ethnicity. One study limited participants to African Americans [40], and two studies included males only [41,42]. Sample sizes ranged from 319 to 260,061.

All studies used multiple data sources. Main data sources for participants and their primary outcomes came from surveys and local, regional, or national representative datasets. For neighborhood attributes, most studies (n = 28) acquired data from local, regional, or national governmental agencies, and 10 studies used private-sector commercial data sources. Instruments used included the Center for Epidemiologic Studies Depression Scale (CES-D) (n = 9); Kessler Psychological Distress Scale (n = 7); Geriatric Depression Scale (GDS) (n = 4); General Health Questionnaire (GHQ) (n = 4); Patient Health Questionnaire (PHQ) (n = 2); Brief Symptom Inventory (BSI) (n = 1); Composite International Diagnostic Interview Short-Form for Major Depression (CIDI-SFMD) (n = 1); Depression Anxiety and Stress Scales (DASS) (n = 1); Mini-International Neuropsychiatric Interview (MINI) (n = 1); Mental Health Inventory (MHI) (n = 1); and the Depression Subscale from the Revised Symptom Checklist (SCL-90-R) (n = 1).

### 3.3. Study Quality

Thirty studies (93.8%) had high methodological quality (met 6–7 criteria), and two studies (6.2%) had moderate methodological criteria (met 4–5 criteria) [43,44] (Table 1). Fifteen studies (50%) met all applicable criteria. Fourteen studies (46.7%) met six of the seven criteria, with the seventh criterion rated as not clear. Inclusion criteria were unclear in two studies and details on study participants and settings were limited in three studies. Most of the studies (n = 25; 83%) used valid or reliable measures of the neighborhood attributes. The reliability or validity of the GIS measurements of neighborhood attributes was unclear in five studies (17%). All studies (97%) except one [43] addressed confounding factors explicitly through the use of statistical adjustments, stratifications, and model selection. All studies used reliable and valid measurements of depressive symptoms or psychological distress. Finally, 25 studies (83%) used appropriate statistical analyses to address their research aims. Five studies (17%) did not provide justification of their analytical approach using model comparisons or checking assumptions.

**Table 1:** Summary of methodological quality of included studies.

Author, Date	Clear Definition of Sample Inclusion Criteria	Study and Setting Described in Detail	Valid and Reliable Measurement of the Exposure	Identification of Confounding Factors	Strategies for Confounding Factors Described	Valid and Reliable Measurement of the Outcomes	Appropriate Statistical Analysis Used	Met Quality Criteria (%)
Ambrey, 2016a	Y	UN	Y	Y	Y	Y	Y	85.7
Ambrey, 2016b	Y	UN	Y	Y	Y	Y	Y	85.7
Annerstedt et al., 2012	UN	Y	Y	Y	Y	Y	Y	85.7
Astell-Burt et al., 2013	Y	Y	Y	Y	Y	Y	UN	85.7
Astell-Burt et al., 2019	Y	Y	UN	Y	Y	Y	Y	85.7
Berke et al., 2007	Y	Y	Y	Y	Y	Y	UN	85.7
Beyer et al., 2014	Y	Y	Y	Y	Y	Y	Y	100
Cromley et al., 2012	Y	UN	Y	N	UN	Y	Y	57.1
DeGuzman et al., 2013	Y	Y	Y	Y	Y	Y	Y	100
Francis et al., 2012	Y	Y	Y	Y	Y	Y	Y	100
Gariepy et al., 2015a	Y	Y	Y	Y	Y	Y	Y	100
Gariepy et al., 2015b	Y	Y	Y	Y	Y	Y	Y	100
Ho et al., 2017	Y	Y	UN	Y	Y	Y	Y	85.7
Ivey et al., 2015	Y	Y	Y	Y	Y	Y	Y	100
Koohsari et al., 2018	Y	Y	Y	Y	Y	Y	UN	85.7
Mayne et al., 2018	Y	Y	Y	Y	Y	Y	Y	100
Moore et al., 2016	Y	Y	UN	Y	Y	Y	Y	85.7
Noordzij et al., 2020	Y	Y	Y	Y	Y	Y	Y	100
Nutsford et al., 2016	UN	Y	Y	Y	Y	Y	Y	85.7
Rantakokko et al., 2018	Y	Y	Y	Y	Y	Y	UN	85.7
Saarloos et al., 2011	Y	Y	Y	Y	Y	Y	Y	100
Sakar et al., 2013	Y	Y	Y	Y	Y	Y	Y	100
Schootman et al., 2007	Y	Y	Y	Y	Y	Y	Y	100
Song et al., 2007	Y	Y	Y	Y	Y	Y	Y	100
Su et al., 2019	Y	Y	Y	Y	Y	Y	Y	100
Thomas et al., 2007	Y	Y	Y	Y	Y	Y	Y	100
Tomita et al., 2017a	Y	Y	Y	Y	Y	Y	Y	100
Tomita et al., 2017b	Y	Y	Y	Y	Y	Y	UN	85.7
Traoré et al., 2020	UN	Y	UN	Y	Y	Y	Y	71.4
van den Bosch et al., 2015	Y	Y	Y	Y	Y	Y	Y	100
Zhang et al., 2018	Y	Y	UN	Y	Y	Y	Y	85.7
Zhang et al., 2019	Y	Y	UN	Y	Y	Y	Y	85.7

Ratings: Y = Yes; N = No; UN = Unclear.

**Table 2:** Characteristics of included studies.

Author, Date	Country	Study Area	Framework	Study Design	Participants	Data Sources (Population)	Data Sources (Neighborhoods)	Outcome Measure
Ambrey, 2016a	Australia	7 major cities ^1^	Empirical evidence	Cross-sectional	6082 age 15+ adults	HILDA	PSMA Australia Limited Transport and Topography dataset	Kessler
Ambrey, 2016b	Australia	7 major cities ^1^	Empirical evidence	Cross-sectional	6077 age 15+ adults	HILDA	PSMA Australia Limited Transport and Topography dataset; Australian Bureau of Statistics	Kessler
Annerstedt et al., 2012/ van den Bosch et al., 2015	Sweden	Scania region	Empirical evidence	Longitudinal cohort study	9230/7549 ^2^ age 18+ adults	Swedish registration system linked survey in a follow-up public health study	The National Land Survey of Sweden (Coordination of Information on the Environment); regional GIS databases; Swedish Environmental Protection Agency; County Administrative Board	Kessler
Astell-Burt et al., 2013	Australia	New South Wales	Empirical evidence	Cross-sectional	260,061 age 45+ adults	45 and Up Study	Australian Bureau of Statistics	Kessler
Astell-Burt et al., 2019	Australia	Sydney; Wollongong; Newcastle	Empirical evidence	Longitudinal cohort study	46,786 age 45+ adults	45 and Up Study	Geovision (Pitney Bowers Ltd.)	Kessler
Berke et al., 2007	USA	King County	Social stress model	Cross-sectional	740 age 65+ older adults	Adult Changes in Thought Study	Walkable and Bikable Communities Project (King County GIS Center)	CES-D
Beyer et al., 2014	USA	Wisconsin	Attention Restoration/Stress Reduction Theory	Cross-sectional	2479 age 21+ adults	Survey of the Health of Wisconsin	Landsat 5 Satellite imagery (USGS); National Land Cover Database	DASS
Cromley et al., 2012	USA	New Jersey	Empirical evidence	Cross-sectional	5554 age 50+ adults	ORANJ BOWL	US Census Bureau; The Uniform Crime Report State of New Jersey Division of State Police Uniform Crime Reporting Unit	CES-D
DeGuzman et al., 2013	USA	San Antonio; Chicago; Boston	Conceptual framework ^3^	Cross-sectional	1697 adults (mean 38 years)	Welfare, Children and Families: A Three City Study	US Census Chicago Transit Authority and VIA Metropolitan Transit; US Census Bureau	BSI
Francis et al., 2012	Australia	Perth	Social-ecological framework	Cross-sectional	1230 age 18+ adults	RESIDential Environments Project	SENSIS	Kessler
Gariepy et al., 2015a	Canada	Quebec	Empirical evidence	Longitudinal cohort study	372 age 18+ diabetic adults	Diabetes Health Study	DMTI Lightbox; Statistics Canada; Satellite imagery (Canadian Council on Geomatics)	PHQ
Gariepy et al., 2015b	Canada	National	Empirical evidence	Longitudinal cohort study	7114 age 18+ adults	National Population Health Survey	DMTI Lightbox	CIDI-SFMD
Ho et al., 2017	China	Hong Kong	Data-driven approach	Cross-sectional	3930 age 65+ older adults	Cohort study	Hong Kong Planning Department; IKONOS multispectral imagery (Satellite imaging corporation)	GDS
Ivey et al., 2015	US	Alameda; Cook; Allegheny; Wake; Curham Counties	Social-ecological framework	Cross-sectional	870 age 65+ adults	Healthy Aging Research Network’s Walking Study	Environmental Systems Resource Institute Business Analyst; US Census Bureau	CES-D
Koohsari et al., 2018	Australia	Melbourne	Social-ecological framework	Cross-sectional	319 age 25+ adults	Australian Diabetes Obesity and Lifestyle Study	VicMap Features of Interest dataset (Department of Sustainability and Environment)	CES-D
Mayne et al., 2018	Australia	Sydney	Empirical evidence	Cross-sectional	91,142 age 45+ adults	45 and Up Study	Census of Population and Housing; Australian Bureau of Statistics; New South Wales Department of Planning and Infrastructure; New	Kessler
Moore et al., 2016	US	Forsyth County; NYC; Baltimore; St Paul; Chicago; LA	Empirical evidence	Longitudinal cohort study	5475 age 45+ adults	Multi-Ethnic Study of Atherosclerosis	South Wales Department of Land and Property Information; Property Council of Australia and City of Sydney Council National Establishment Time Series database (Walls & Associates)	CES-D
Noordzij et al., 2020	Netherlands	Eindhoven	Psycho-evolutionary theory	Longitudinal cohort study	3175 age 15+ adults	GLOBE	Bestand Bodemegruik (Statistics Netherlands)	MHI
Nutsford et al., 2016	New Zealand	Wellington	Empirical evidence	Cross-sectional	442 age 15+ adults	New Zealand Health Survey	Land Class DataBase II; Department of Conservation land register; Land Information New Zealand parcel database; Land Information New Zealand (LINZ)	Kessler
Rantakokko et al., 2018	Finland	Central Finland	Empirical evidence	Cross-sectional	848 age 75+ adults	GEOage Project; Life-space mobility in old age Project	Finnish Environment Institute	CES-D
Saarloos et al., 2011	Australia	Perth, Western Australia	Empirical evidence	Cross-sectional	5218 65+ male adults	Health in Men Study	Western Australia Department for Planning and Infrastructure; Australian Bureau of Statistics	GDS
Sakar et al., 2013	UK	Caerphilly, South Wales	Empirical evidence	Cross-sectional	687 age 65+ male adults	Caerphilly Prospective Study	UK Ordnance Survey Master Map dataset; Landsat 7 dataset (USGS); UK Office of National Statistics	GHQ
Schootman et al., 2007	US	St Louis, MO	Social disorganization theory	Longitudinal cohort study	998 middle-age African Americans	African American Health Study	US Census Bureau	CES-D
Song et al., 2007	US	LA	Stress process in neighborhood context	Cross-sectional	1503 age 18+ adults	Chinese American Psychiatric Epidemiologic Study survey	US Census Bureau; LA GIS center	SCL-90-R
Su et al., 2019	Spain	Barcelona	Empirical evidence	Cross-sectional	3461 age 18+ adults	2011 Barcelona Health Survey	WorldView2 imagery (DigitalGlobal); RapidEye imagery (RapidEye AG); Landsat8 imagery (USGS)	GHQ
Thomas et al., 2007	UK	Neath Port Talbot County Borough, South Wales	Empirical evidence	Cross-sectional	1508 age 16+ adults	Housing And Neighborhood And Health	Neath Port Talbot County Borough Council	GHQ
Tomita et al., 2017a	South Africa	National	Empirical evidence	Longitudinal cohort study	11,156 age 15+ adults	SA-NIDS	National Aeronautics and Space Administration MODIS satellite (MOD13A3)	CES-D
Tomita et al., 2017b	South Africa	KwaZulu-Natal Province	Behavioral Model of Health Services Use framework	Longitudinal cohort study	4309 age 15+ adults	SA-NIDS	KZN Department of Health	CES-D
Traoré et al., 2020	France	Paris	Empirical evidence	Cross-sectional	3006 age 15+ adults	SIRS	INSEE	MINI
Zhang et al., 2018/2019	China	Hong Kong	Social-ecological model	Cross-sectional	909 age 65+ adults	Active Lifestyle and the Environment in Chinese Seniors Project	Census and Statistics, Lands, and Planning Department of HKSAR	GDS

Note. Explanations of acronyms are in alphabetical order: BSI, Brief Symptom Inventory; CES-D, Center for Epidemiologic Studies Depression Scale; CIDI-SFMD, Composite International Diagnostic Interview Short-Form for Major Depression; DASS, Depression Anxiety and Stress Scales; GDS, Geriatric Depression Scale; Geographic characteristics, outdoor mobility, and physical activity of older people (GEOage) Project; GIS, Geographic Information Systems; GLOBE, Gezondheid en Levens Omstandigheden van de Bevolking van Eindhoven en omstreken; HILDA, Household, Income and Labour Dynamics in Australia Study; INSEE, French National Institute for Statistics and Economic Research; KZN, KwaZulu-Natal; HKSAR, Hong Kong Special Administrative Region; LA, Los Angeles; MHI, mental health inventory; MINI, Mini-International Neuropsychiatric Interview; MO, Missouri; NYC, New York City; ORANJ BOWL, Ongoing Research on Aging in New Jersey—Bettering Opportunities for Wellness in Life; PHQ, Patient Health Questionnaire; SA-NIDS, South African National Income Dynamics Study; SCL-90-R, Depression subscale from the Revised Symptom Checklist; SIRS, a French acronym for “health, inequalities and social rupture”; UK, United Kingdom; US, United States; USGS, United States Geological Survey. ^1^ Adelaide, Brisbane, Canberra, Darwin, Melbourne, Perth and Sydney; ^2^ Persons who have moved were excluded from the cohort in van den Bosch et al.’s (2015) study; ^3^ Framework links the built environment to health outcomes through social and economic conditions, social support, and stressors.

### 3.4. Neighborhood Attributes and Geographical Unit

Neighborhood attributes measured by GIS are summarized in Table 3. The most common attributes studied were environmental (n = 16); proximity (n = 10); infrastructure (n = 8); residential characteristics (n = 6); and social and demographic neighborhood attributes (n = 8). Twenty-one studies (65.6%) focused on single neighborhood attributes as independent variables, with 11 studies (34.4%) including multiple neighborhood attributes.

Ten studies (31%) used administrative/statistical geographical units to measure neighborhood attributes: census collection district (n = 4), census tract (n = 3), block group (n = 4), and postal area (n = 1). Twenty studies (63%) used person-centered unit (buffers) (n = 17) and distance-based measurements (n = 3). Two studies used both buffers and distance-based measurements [24,45]. One study [40] compared two different administrative/statistical units. Traoré et al.’s [44] study included residential, work-place, and frequented administrative/statistical units to measure outcomes. Eight studies used the buffer size of ≤500 m area as a geographical unit. Five studies used the buffer size of >500 m and ≤10,000 m area. Eight studies compared the relationships between neighborhood attributes and outcomes at multiple geographical units, and only two studies reported the optimal results on a specific unit [25,46].

The construction of GIS-derived neighborhood attributes varied across studies even when measuring the same neighborhood attribute. First, green spaces were measured most frequently as environmental neighborhood attributes. Neighborhood attributes measuring environmental characteristics were constructed using diverse GIS methods. The exception was the NDVI (Normalized Difference Vegetation Index), which was used in six studies. NDVI was calculated on grids using raster data and this index was aggregated on either administrative/statistical neighborhood unit or buffer areas created by GIS. One study [36] used machine learning techniques to capture green spaces in image data.

Neighborhood resource characteristics were measured by the proximity to destinations of restaurants, businesses, or physical activity-related facilities within a certain neighborhood geographical unit [25,26,47]. Circular buffer areas or network buffer areas were used to aggregate the total numbers of neighborhood resources within those areas, and multiple buffers were used to test the significance of the neighborhood geographical unit to explain depressive symptoms or psychological distress. Infrastructure characteristics include the concepts of walkability or street connectivity. The frequently used components to create a composite index of walkability or street connectivity included streets or land-use. Residential characteristics were measured by aggregating the housing units in administrative/statistical areas.

Social and demographic characteristics were measured by aggregated values within administrative/statistical neighborhood units. Social and demographic characteristics were measured in the area-based composite variables using multiple factors (e.g., income level, old population, educational attainment) based on certain concepts (e.g., neighborhood deprivation).

### 3.5. Use of Geographic Information Systems

Summaries of the GIS used by the study design stage are described in Table 4. For participants, four studies used spatial sampling techniques based on administrative/statistical geographical units. One study [23] used the geocoded data of potential participants to select a geographically random sample.

For data acquisition, data types for neighborhood attributes included point, line, and/or polygon data (n = 11), topological data (e.g., land cover data) (n = 8), multiple datasets (n = 7), image data from the satellite (n = 3), and administrative data (n = 3). One study [48] compared image data from different sources using remote sensing techniques. For data preparation, GIS measurement functions of an area or the volume were frequently used alone or with other functions such as distance or length [49]. The buffering measurement function was used to define the person-centered geographical unit in 18 studies. Radial buffering was used more often than network buffering. Only two studies used surface analysis to measure environmental characteristics [42,50]. For data analysis, eight studies used GIS for an exploratory analysis within a geographical unit using administrative data such as census data. One study used global and local spatial autocorrelation, geostatistics, and spatial weights for modeling. For data presentation, mapping was used to represent the neighborhood attributes (n = 7) and statistical estimates (n = 3); two studies used mapping to compare datasets [48] or to present outcomes [44].

For the data management of participants or neighborhood attributes, addresses were transformed to geographic coordinates, which is called geocoding. Two studies used GPS (Global Positioning System) coordinates for the participants’ households. Geographic data were divided by vector data (discrete data) and raster data (continuous data). The most used GIS function was overlay. The overlay function linked the aggregated/calculated neighborhood attributes in types of vector or raster data on a neighborhood geographical unit. The spatial join was used to link the geographical units with neighborhood attributes. For image analysis, zonal statistics or raster-to-vector or vector-to-raster conversion were used.

### 3.6. Association of Neighborhood Attributes with Mental Health Outcomes

Table 5 summarizes the studies with significant relationships between neighborhood attributes and psychological distress or depressive symptoms. Significant neighborhood attributes were: environmental (% green space, % tree canopy, blue space visibility, slope variability, green exposure, and nearest green space; green or blue space; green or agricultural space; green, blue or agricultural space), residential (% of residential areas, the variation of building heights, the average building height), sociodemographic (neighborhood poverty, residential stability, residential income level, and cumulative exposure to deprivation), public services (crime), infrastructure (land-use mix), and proximity characteristics of neighborhood resources (proximity to the nearest primary healthcare clinic). Significant moderators existed in explaining the relationships between neighborhood attributes and outcomes. These included: infrastructure (accessibility of streets, land-use availability, land-use configuration, land-use mix, major street, and walkability), environmental (access to green qualities, green exposure, green parkland ratio), sociodemographic (neighborhood deprivation, vehicle burden), neighborhood resources (neighborhood resources, social engagement destinations), and residential characteristics (dwelling level configuration). Supplementary Table S1 presents details of significant relationships between neighborhood attributes and mental health outcomes in the final models adjusted by individual and/or neighborhood confounders.

Residential/non-residential characteristics. Living in houses with a terrace was associated with lower odds of psychological distress in male older adults within a 1 km network buffer [42]. Within a 400 m radial buffer, higher variation in building heights and percentage of residential areas was associated with increased depression risk. In contrast, a higher average building height was associated with decreased depression risk in older adults [51].

Infrastructure characteristics. Greater land-use mix, local-level streets (access to destinations within 1200 m areas by walking) were associated with a decreased risk of psychological distress, whereas a higher density of businesses, offices, and bus stops was mildly associated with the risk of psychological distress in male older adults within a 1 km network buffer [42].

Significant moderating effects, but not main effects, existed (n = 3). Living in areas with more diverse land-use and with retail was associated with increases in depressive symptoms in male older adults within a census collection district [41]. The association of perceived traffic stress and depressive symptoms was associated with increases for adults living in areas with major streets within a block group [52]. Higher walkability within the neighborhoods was associated with decreased risks of depressive symptoms in male but not in female older adults within a 100 m, 500 m, and 1000 m radial buffer [53].

Social and demographic characteristics. Eight studies examined sociodemographic characteristics related to outcomes but used different geographic units and analytics, yielding inconsistent results. Higher risks of psychological distress in male older adults were slightly associated with living in areas with lower employment deprivation and higher physical environment deprivation calculated within a Lower Super Output [42]. A higher poverty level was associated with increased depressive symptoms for adults in 80% of the census tracts in New Jersey. Higher residential stability was associated with decreased depressive symptoms for adults in the northern part of New Jersey [43]. The effect of deprivation on increasing the risks of depressive symptoms in diabetic adults was stronger in older and retired individuals within a 500 m radial buffer [46]. The association between perceived traffic stress and depressive symptoms was related to increases in depressive symptoms for adults living in areas with a greater vehicular burden within a block group [52]. Individuals residing in poor or average income-level areas and people who frequented different types of neighborhoods or poor neighborhoods reported significantly higher levels of depressive symptoms [44]. The crime rate was significantly related to depressive symptoms (n = 3). For example, a higher crime rate per 1000 adults was associated with increases in depressive symptoms in 60% of the census tracts in New Jersey [43].

Environmental characteristics. As neighborhood protective resources, 15 studies (50%) included greenness related to outcomes, which presented inconsistent results by population, GIS measurement, or geographical unit. A lower risk of psychological distress in middle-aged and older adults was associated with living with more green spaces within a 1 km radial buffer [35]. One study [35] found significant associations of exposure to more tree canopy with a lower prevalence and incidence of psychological distress in middle-aged and older adults within a 1.6 km network buffer. However, this association was inconsistently found in examinations of relationships between the proportion of green spaces and grass and the prevalence and/or incidence of psychological distress [36]. Living in areas with a higher visibility of blue space >3 km was associated with decreases in psychological distress in adults [50]. Higher risks of psychological distress in male older adults were associated with living in areas with more slope variability within a 1 km network buffer [42]. A higher green exposure (NDVI) level was associated with decreasing the risk of psychological distress at 50 m, 100 m, 250 m, and 500 m radial buffers [48]. Living in 25% higher levels of green spaces, higher green exposure (NDVI), and more tree canopy within a block group was associated with decreases in depressive symptoms in adults [54]. Distances to the nearest green space, blue space, or agricultural space were significantly related to lower depressive symptoms at baseline, but changes in them over 10 years were not significantly related [45].

No significant main effects of environmental attributes were found with either mental health outcomes. Significant moderating effects were found, although they varied between studies. Green space was linked more to lower levels of psychological distress in adults living in more populated neighborhoods who engage in physical activity within a census collection district [32]. Access to green qualities (serene or spacious green spaces) was significantly associated with a decreased risk of poor mental health in women but not in men [33,34]. The association of perceived traffic stress and depressive symptoms was less strong for adults living in areas with a higher green parkland ratio within a block group [52]. Living with higher green exposure at a 1 km distance in middle-income adults was associated with decreases in depressive symptoms compared to low-income adults [38].

Neighborhood resource characteristics. Eight studies focused on proximity characteristics of neighborhood resources. Living in areas with more physical activity facilities and cultural services was associated with decreasing the risk of depressive symptoms in adults with diabetes within a 500 m radial buffer [46]. The presence of any parks, healthy food stores, fast food restaurants, or health services was associated with decreasing the probability of having depressive symptoms in adults at a 500 m radial buffer [55]. The presence of any parks was also associated with a moderate probability of having depressive symptoms [55]. Adults living in areas more than 6 km away from a primary health care clinic reported significantly higher levels of depressive symptoms than adults living in areas with a primary health clinic within 6 km [39].

Three studies reported significant moderating effects. Living in areas with greater social engagement facilities was associated with decreasing depressive symptoms in middle-aged and older females but not in males within a 1 mile radial buffer. This effect was significant at baseline, but no significant changes were found at 10-year follow-up [56]. Living in areas with poorer access to civic/institutional destinations, retail, food/eating outlets, public transport stops, and health clinics/services was significantly associated with an increase in depressive symptoms in older adults who live alone within an 800 m radial buffer [25]. Better access to neighborhood resources (civic/institutional destinations, retail, food/eating outlets, and health clinics/services) was associated with a higher frequency of walking for transport in older adults living alone, but not in those living with others. A higher frequency of walking for transport was negatively related to lower levels of depressive symptoms, but better access to destinations was not directly related to outcomes [26].

## 4. Discussion

This is the first systematic review to describe the applications of GIS methods in mental health research and the relationships between objectively measured neighborhood attributes and depressive symptoms and psychological distress in adults. The results from 32 high- and moderate-quality studies confirm and extend previous research that neighborhood attributes objectively measured by GIS are important social determinants of mental health.

### 4.1. Use of GIS in Measuring Neighborhood

GIS has been used successfully to objectively measure a variety of physical and sociodemographic neighborhood attributes. These included physical neighborhood characteristics such as environmental (e.g., green spaces), infrastructure (e.g., walkability), residential (e.g., housing units), building heights, and proximity to neighborhood resources (e.g., service facilities). GIS was also used to measure sociodemographic neighborhood characteristics such as crime. Those neighborhood attributes were found to be significantly related to depression in prior review papers and the current review paper identified these neighborhood attributes used by GIS. GIS was frequently used to measure natural neighborhood attributes or physical infrastructural neighborhood attributes. Social and demographic neighborhood attributes were collected on the neighborhood level from governmental agencies, which enables GIS to easily map the socioeconomic neighborhood attributes. However, Galster’s [27] neighborhood attribute categories of public service, political, social-interactive, and emotional characteristics were not examined in relationship with mental health outcomes in the selected studies.

### 4.2. GIS-Derived Measurements

GIS has been incorporated into every study phase, starting with population recruitment for spatial sampling based on geocoded data [23] to the visualization of statistical results [43]. Most frequently, GIS was used to create the measurement of neighborhood attributes as independent variables to examine their relationships with outcomes of depression. Although different neighborhood attributes measured by GIS were used across studies, common capabilities of GIS were found in defining “neighborhoods” and measuring “neighborhood attributes.” Prevalent GIS methods used involved aggregating neighborhood attributes coded in certain areas. The administrative or statistical neighborhood units were the most frequently used unit to aggregate sociodemographic neighborhood characteristics. To quantify physical environmental characteristics (e.g., neighborhood resources), person-centered neighborhood units (e.g., buffer areas) need geocoding processing. Different methods of buffer analysis, such as “Network,” account for the infrastructure, such as roads or sidewalks, to define the neighborhood in terms of accessibility [21]. Geocoded data link multiple datasets with survey, local, governmental, or image data, and these data sources came from more diversified origins such as local/national governmental agencies to commercial data.

Among physical neighborhood characteristics, environmental characteristics including natural features were captured most effectively by using GIS. Some studies used surface analysis to measure topological characteristics [42,50] or used fine image data [48]. The GIS techniques were diversified in measuring environmental neighborhood attributes in terms of types of data or spatial arithmetic operations. For instance, to measure the concept of greenness, the total areas of green spaces were aggregated within a geographical unit [31,32], while vegetation cover on grids was calculated to derive green spaces by certain characteristics such as places of peace or wild nature [33,34]. To find significant neighborhood attributes to aggregate the natural features, one study used a sensitivity analysis to compare image data linked to multiple datasets to evaluate data reliability [48]. The visualization of green spaces via mapping can present spatial patterns of neighborhood attributes, identify vulnerable places, and provide estimates of spatial statistical analysis quantifying these patterns [37,42,44,51].

Neighborhood resource characteristics were frequently aggregated on buffer areas. Because an address is point data, GIS can calculate the total number of points within a neighborhood unit. Circular or network buffer areas were created using GIS as neighborhood units. The strength of this neighborhood unit is flexibility to set the distance from the participant’s home; however, these types of neighborhood unit cannot be adjacent to each other by the boundary.

Infrastructure characteristics were measured by a composite variable measured by diverse features in the neighborhood such as street, intersection, or land-use. For example, walkability is an index invented to quantify the walkable neighborhood considering infrastructure characteristics. Individual infrastructural attributes may not describe the landscape of the neighborhood to explain the health outcomes; however, the composite variables of neighborhood attributes can explain more about the mental health outcomes.

Social and demographic neighborhood attribute data were provided in certain administrative/statistical neighborhood units such as census tracts because location data can be used to identify a specific individual. These aggregated neighborhood-level data were publicly accessible and convenient for use. However, a limitation of these aggregated data is that they are not modifiable to a different neighborhood unit such as buffer areas beyond the predetermined administrative/statistical neighborhood unit. Data sources to construct neighborhood attributes came from local or national governmental agencies related to neighborhood-level socioeconomic characteristics. The strength of administrative/statistical neighborhood units was determined by local or national governments so that data collection is reliable with the systematic survey process. Administrative/statistical neighborhood units are adjacent by the boundaries, so spatial analysis can be conducted in large geographical areas. Spatial data analysis revealed significant geographical differences between neighborhood attributes of poverty or crime and depression [43].

### 4.3. Neighborhood Definitions

The definition of neighborhood was inconsistent across studies. There were strengths and limitations with the diverse neighborhood definitions. Almost all studies used the definition of neighborhood around the home or participants. A recent study used different types of neighborhoods, dividing by residential, work, and frequented areas [44].

Data availability also determines the neighborhood definition so that it may not have the “best” neighborhood in terms of defining neighborhood boundaries. Two representative neighborhood geographical units included administrative/statistical geographical neighborhood units (e.g., census tract) and buffer areas created by GIS (e.g., 800 m-circular buffer areas). Administrative/statistical geographical units are representatives of neighborhoods with reasonable homogeneity in population size (e.g., census tract), and they are frequently used for demographic data for administrative purposes such as in the American Community Survey, which is the yearly national survey by the U.S. Census Bureau. Buffer areas created by GIS are person-centered geographical units that are measured based from/to a resident’s home to/from a certain destination. GIS allows for the definition of neighborhoods for each resident, which constitutes the concept of person-centered neighborhoods [57]. This review was unable to identify the optimal buffer area around neighborhoods that were related to mental health outcomes. Some studies used sensitivity analysis testing with different buffer sizes to find the best fit for models statistically [24,25,46,48,53].

### 4.4. Effects of GIS-Derived Neighborhood Attributes on Mental Health

Consistent with prior reviews, this review found that socioeconomic composition, social processes [20], built environment, and residential environment are related to depression [41,43,51] in general. By specific neighborhood attributes, findings suggested the inconsistent results regarding significance association between neighborhood attributes measured by GIS and mental health outcomes. For example, one study showed the significance relationship of green spaces with psychological distress [48]; however, one study did not [50]. This may be because of differences in neighborhood geographical units, GIS methods to measure neighborhood attributes, or interactions of multi-layered or complex neighborhood attributes.

The natural environment of green or blue spaces and access to primary healthcare clinics were also related to depression [35,36,39,45,48,50,54]. However, results were inconsistent when examining the same neighborhood attributes. For example, green exposure measured by NDVI was not related to depressive symptoms within a 500 m buffer [46,58] or a 400 m buffer [51], but was related in a block group [54]. This is because the variances at different spatial scales may lead to different interactions between the neighborhood attributes and mental health outcomes within areas [59].

Some significant GIS-derived neighborhood attributes were related to depression; however, the majority were not. This might be explained by the complex relationships among neighborhood attributes and mental health as well as factors that could moderate the effects such as demographic characteristics, socioeconomic status, living arrangement, and residential environment. For instance, women perceive nature or places that build social support in their communities more importantly than men [60]. Additionally, older or retired adults may be more influenced by their neighborhoods because of their physical morbidity or limitations [61]. With known protective effects of nature on mental health, physically active adults or adults with middle incomes benefited more from green spaces than others [35,38]. In populated areas (e.g., vehicle-dense areas), green spaces provided more protective effects on negative mental health outcomes [62].

The associations of neighborhood attributes with mental health outcomes were inconsistent across studies. For example, Cromely et al., [43] reported a significant association of living in areas having higher poverty levels with higher levels of depressive symptoms for adults middle-aged and older in 80% of the study areas. In contrast, Ivey et al., [23] did not find that living in a deprived neighborhood significantly increased the odds of depressive symptoms for older adults. This inconsistency might be explained by the varied distributions of mental health outcomes in different areas or in different age groups. Neighborhood-level socioeconomic status was measured in an aggregated manner, lacking individual variations that could potentially explain mental health outcomes measured on the individual level [63].

### 4.5. Strength and Limitations

The strengths of this systematic review include that it is the first to synthesize the evidence on the use of GIS in measuring neighborhood attributes and how GIS-derived neighborhood attributes are associated with mental health outcomes in adults. The review incorporated a comprehensive search of diverse databases that yielded 32 moderate- and high-quality studies. The limitations of this review are the inclusion of studies published in English only, the lack of consistent neighborhood attributes examined, and methodological weaknesses in individual studies that made it difficult to derive definitive conclusions. These weaknesses included the use of cross-sectional designs, self-report measures of mental health, variations in GIS measurements of neighborhood attributes and defined geographical units. In particular, the Modifiable Area Unit Problem (MAUP) may be present when environments and participants are analyzed at different geographic scales, which produces significant variations in research results. Another limitation was the lack of studies located in small or rural areas.

### 4.6. Future Research

Future studies are needed to evaluate GIS-derived neighborhood measures and their relationship to mental health outcomes in diverse populations. Research is needed that focuses on specific demographic factors or targets individuals with specific mental health conditions. Studies conducted in large areas should examine spatial estimates to identify geographical differences or consider spatial weights to adjust for those differences. The neighborhood units or GIS techniques found from this review can be re-tested to identify the neighborhood attributes that are significantly related to mental health outcomes.

## 5. Conclusions

This review demonstrates how GIS can measure physical neighborhood attributes objectively and expand the scope of neighborhood-related mental health research. The results indicate how GIS-derived neighborhood measurements can be used when examining the social determinants of depressive symptoms and psychological distress in adults in terms of physical environmental characteristics. Person-centered neighborhood units created by GIS as well as administrative neighborhood units should be used based on the study purpose, data availability, and/or neighborhood attributes of interest to measure. Because of the complexity of neighborhood-related mental health research, study results should be carefully interpreted, with consideration given to potential moderating factors such as demographic characteristics. GIS methods are still being developed. However, researchers should consider using neighborhood geographical units or GIS-derived measurements as they offer a valuable method to examine neighborhood impacts on mental health. The standardization of the neighborhood unit or GIS-derived measures of neighborhoods may be needed in order to explain depression or psychological distress for the comparison of results across studies. Future studies are needed to evaluate GIS-derived neighborhood measures and their relationship to mental health outcomes in diverse populations that vary by age, race/ethnicity, etc.

## Figures and Tables

**Table 3:** Neighborhood attributes, measurements, and geographical units.

Author, Date	Attributes	Measurement Details	Geographical Unit
Environmental Characteristics			
Ambrey, 2016a; 2016b	Green spaces	- Hectares per capita or square kilometers per CCD of greenspace (public parks, community gardens, cemeteries, sports fields, national parks, and wilderness areas)	Census Collection District
Annerstedt et al., 2012; van den Bosch et al., 2015	Green qualities	- Assessment of the presence of green qualities (Serene, Wild, Lush, Spacious, and Culture) ▪ Serene: a place of peace, silence, and care ▪ Wild: a place of fascination with wild nature ▪ Lush: a place rich in species ▪ Spacious: a place offering a restful feeling of “entering another world” ▪ Culture: the essence of human culture	300 m radial buffer
Astell-Burt et al., 2013	Green spaces	- % Sum of parks, woodland, bush, other vegetation areas	1 km radial buffer
Astell-Burt et al., 2019	Green spaces	- % Total green space - % Tree canopy - % Grass - % Low-lying vegetation	1.6 km network buffer
Beyer et al., 2014	Green spaces	- Green exposure: Normalized difference vegetation index (NDVI) - % Tree canopy coverage - Green space: NDVI and tree canopy average	block group
Gariepy et al., 2015a	Green exposure	- Normalized difference vegetation index (NDVI)	500, 1000, 1500 m radial buffers
Ho et al., 2017	Vegetation	- % Vegetation using normalized difference vegetation index (NDVI)	400 m radial buffer
Noordzij et al., 2020	Green exposure	- % Green space - Distance to nearest green spaces; green or blue spaces; green or agricultural spaces; green, blue or agricultural spaces	300, 500, 1000 m radial buffer
Rantakokko et al., 2018	Nature diversity	- Shannon Diversity Index (SHDI): Natural environment (cultivated fields, fruit trees and berry plantations, pastures, uncultivated agricultural areas, forests, shrub, and/or barbaceous vegetation, open spaces with little/no vegetation)	500 m radial buffer
Sakar et al., 2013	Green exposure Slope variability	- Normalized difference vegetation index (NDVI) - Degree of variability in slope	500 m radial buffer 1 km network buffer
Song et al., 2007	Green parkland ratio	- Percent of park land area	block group
Su et al., 2019	Greenness exposure	- Normalized difference vegetation index (NDVI)	50, 100, 250, 500 m radial buffers
Tomita et al., 2017a	Greenness exposure	- Normalized difference vegetation index (NDVI)	1 km resolution grid
**Environmental Characteristics**			
Nutsford et al., 2016	Visibility of green and blue spaces	- Vertical Visibility Index (VVI): accounts for the slope, aspect, distance and elevation of visible areas relative to the observer’s location, a visual summation of green and blue spaces in degrees of visibility	<300 m, 300 m–3 km, 3–6 km, 6–15 km from centroids of meshblocks
**Infrastructure Characteristics**			
Berke et al., 2007	Walkability	- Average walkability score within the buffer; distance to parks, foot trails, bicycle trails, land slope, and public transit	100, 500, 1000 m radial buffers
DeGuzman et al., 2013	Public transportation	- Distance to public transportation (train and bus stops)	Not applicable
Mayne et al., 2018	Walkability	- Composite index: Residential dwelling density; intersection density; land-use mix	Postal area
Saarloos et al., 2011	Walkability	- Composite index: Street connectivity; residential density; land-use	Census Collection District
	Street connectivity	- Number of intersections	
	Land-use mix	- Diversity of land uses in an area	
	Land-use availability	- Retail - Other retail - Offices/business - Health/well-being/community services - Entertainment/recreation/culture	
Sakar et al., 2013	Physical accessibility	- Street movement potential	1200, 3000, N-m
	Street connectivity	- Number of segments connected to a segment	1 km network buffer
	Land-use configuration	- Land-use mix score: Residential dwellings, retail, community services, businesses and offices, and recreation and leisure; Density of bus stops, retail, community services recreation and leisure facilities, businesses and offices	
Song et al., 2007	Internal connectivity Major street Land-use diversity	- Number of street intersections divided by the number of intersections plus the number of cul-de-sacs - Length of major street in feet per acre - A diversity index with the distribution of land uses	block group
Zhang et al., 2018; 2019	Street intersection density	- Number of intersections per square kilometers	400, 800 m network buffers
**Residential Characteristics**			
Ho et al., 2017	Environmental measures	- % Residential area - Average building height - Variation of building height	400 m radial buffer
**Residential Characteristics**			
Saarloos et al., 2011	Residential development density	- Average density of residential developments	Census Collection District
Sakar et al., 2013	Dwelling level configuration	- Dwelling-centered density; Dwelling types (detached, semi-detached, terraced, flats); Plot exposure (the number of faces of a dwelling unit exposed to public space)	30 m kernel surrounding
Song et al., 2007	Residential density	- Number of housing units per acre	block group
Zhang et al., 2018; 2019	Residential density	- Number of households per square kilometer	400, 800 m network buffers
**Proximity Characteristics**			
Francis et al., 2012	Quantity of POS	- Number/size of public spaces (parks, recreational grounds, sports fields, commons, esplanades and bushland/wilderness)	1600 m network buffer
Gariepy et al., 2015a	Neighborhood resources	- Density of businesses (health services, physical activity facilities, healthy food stores, fast food restaurants and cultural services (museums, libraries, and botanical gardens), parks and recreational facilities (parks and sports tracks)); Density of express highways; Land-use patterns (land-use mix)	500, 1000, 1500 m radial buffers
Gariepy et al., 2015b	Neighborhood resources	- Presence of any park, healthcare service, healthy food store, fast food restaurant, or cultural service	500 m radial buffer
Ivey et al., 2015	Neighborhood businesses	- The count of business destinations (supermarkets, pharmacies, salons, barber shops, health clubs, gyms, restaurants, coffee shops, banks, theaters, churches, libraries, senior centers)	400 m radial buffer
Koohsari et al., 2018	Quantity of POS	- Network distance between each participant’s home and POS - The size of the nearest POS - The total number of POS within buffers - The total areas of POS within buffers	200, 400, 800, 1000, 1600 m network buffers
Moore et al., 2016	Social engagement destinations	- Density of destinations: participatory entertainment and physical activity (gyms, yoga, bowling, golf); cultural/intellectual (theaters, libraries, museums/galleries, social/political clubs); spiritual/religious (churches, synagogues, mosques); beauty salons and barbers, gambling or coin operated entertainment (casinos, arcades)	1 mile buffer
**Proximity Characteristics**			
Thomas et al., 2007	Geographical accessibility score	- Category 1: nearest bus stop, local shop, pharmacy - Category 2: general practice, post office, cycle path, primary school, children’s play park - Category 3: playing field, public house, supermarket, community center, children’s nursery, bus station, secondary school, train station, swimming pool, sports center, restaurant - Category 4: cinema, non-food stores, bowling green, tennis courts	1: <300 m, 300–500 m, >500 m; 2: <600 m, 600–800 m, >800 m; 3: <800 m, 800–1900 m, >1900 m; 4: <1300 m, 1300–1900 m, >1900 m
Tomita et al., 2017b	Primary Healthcare Clinic	- The ellipsoidal distance to the nearest primary healthcare clinic	6 km radial buffer as a threshold
Zhang et al., 2018; 2019	Neighborhood resources	- Number of parks, density of civic/institutional, retail, entertainment, recreation, food-related (eating outlets), public transport stops	400, 800 m network buffers
**Social and Demographic Characteristics**			
Cromley et al., 2012	Poverty Residential stability	- % Population below the poverty level - % Population who had been living in their present living arrangements for 5+ years	census tract
	Crime	- Total number of major offences (murder, rape, robbery, aggravated assault, burglary, larceny theft, motor vehicle theft)	
DeGuzman et al., 2013	Residential density	- % Population in residence	block group
Gariepy et al., 2015a	Neighborhood deprivation	- Pampalon index: material and social deprivation	census block
Ivey et al., 2015	Neighborhood socioeconomic status	- Composite index: % adults older than 25 years of age; % less than a high school education; % male unemployment; % households with income below the poverty line; % households receiving public assistance; % households with children that are headed by a female; median household income	census tract
Sakar et al., 2013	Area-level deprivation	- Welsh index of multiple deprivation domains: income; employment; health; education; housing; access to services; physical environment	Lower Super Output Area
Shootman et al., 2007	Deprivation index	- Composite variable: % below poverty; % public assistance; % age ≥ 25 years with less than a high school education; % housing units lacking plumbing; % African American race and unemployment rate; % residing for ≥5 years and owner-occupied housing; % female-headed households; percentage aged > 64 years	census tract, block group
Song et al., 2007	Neighborhood poverty Vehicle burden	- % Persons meeting the federal poverty threshold - % Residents aged 16 years or older who drive alone to work	block group
**Social and Demographic Characteristics**			
Traoré et al., 2020	Income level	- Average income per consumption unit (low, average, high)	residential census block, workplace census block, frequented census block
	Cumulative exposure to deprivation	Group 1: Poor neighborhoods only Group 2: Wealthy neighborhoods only Group 3: Neighborhoods of different types	

Note. CCD, Census Collection District; m, meters; POS, public open spaces.

**Table 4:** Geographic information systems used in selected studies by study design stage.

	Data Acquisition	Data Preprocessing			Data Analysis	Data Presentation
	Neighborhood	Measurement				
		Neighborhood Attribute	Neighborhood Unit	Participant		
Ambrey (2016a)	topological data	area	admin	admin		
Ambrey (2016b)	topological data; admin data	area	admin	admin		
Annerstedt et al., (2012)	topological data	area	buffering (radial)	geocoding		
Astell-Burt et al., (2013)	topological data	area	buffering (radial)	centroid (meshblock)		mapping: neighborhood attribute
Astell-Burt et al., (2019)	line data; image data	area	buffering (network)	centroid (meshblock)		
Berke et al., (2007)	point, line data		buffering (radial)	geocoding		mapping: neighborhood attribute
Beyer et al., (2014)	topological data; image data	area	admin	geocoded (address)		mapping: neighborhood attribute
Cromley et al., (2012)	admin data		admin	admin	exploratory data analysis, global/local spatial autocorrelation, geostatistic, spatial weights	mapping: estimate
DeGuzman et al., (2013)	point data; admin data	distance	admin	centroid (block group)		
Francis et al., (2012)	point, line data	volume	buffering (network)	geocoding		
Gariepy et al., (2015a)	image data; point, line data; admin data	area, volume, length	buffering (radial)	centroid (postal code)		
Gariepy et al., (2015b)	point data	volume	buffering (radial)	centroid (postal code)		
Ho et al., (2017)	image data;admin data	area	buffering (radial)	geocoding	exploratory data analysis	mapping: estimate
Ivey et al., (2015)	point data; admin data	volume	buffering (radial)	geocoding		
Koohasari et al., (2018)	point, line, polygon data	distance, volume	buffering (network)	geocoding		
Mayne et al., (2018)	topological data;line data	volume		geocoded (statistical division/postal code)	exploratory data analysis	mapping: estimate
Moore et al., (2016)	point data	volume	buffering (radial)	geocoding		
Noordzij et al., (2020)	topological data	distance, volume	buffering (radial)	geocoding		mapping: neighborhood attribute
Nutsford et al., (2016)	topological data	area		centroid (meshblock)		mapping: neighborhood attribute
Rantakokko et al., (2018)	topological data	area	buffering (radial)	geocoding		
Saarloos et al., (2011)	point, line data; admin data	volume, length		geocoded (statistical division)		
Sakar et al., (2013)	topological data;image data; point, line data;admin data	area, volume, distance	buffering (radial and network)	geocoding	exploratory data analysis	mapping: estimate
Schootman et al., (2007)	admin data			geocoded (statistical division)		
Song et al., (2007)	point, line, polygon data; admin data	area, volume, length		geocoded (statistical division)		
Su et al., (2019)	image data	area	buffering (radial)	geocoding		mapping: study location, comparison of datasets
Tomas et al., (2007)	point data	distance		admin		
Tomita et al., (2017a)	image data	area		GPS coordinate (household)		
Tomita et al., (2017b)	point data	distance		GPS coordinate (household)		mapping: neighborhood attribute
Traoré et al., (2020)	admin data			geocoded (statistical division)		mapping; outcome attribute
van den Bosch et al., (2015)	topological data	area	buffering (radial)	geocoding		mapping: neighborhood attribute
Zhang et al., (2018)	point, line data; admin data	volume	buffering (network)	geocoding		
Zhang et al., (2019)	point, line data; admin data	volume	buffering (network)	geocoding		

Note. GPS, Global Positioning System. Admin, administrative.

**Table 5:** Neighborhood attributes significantly related to mental health outcomes.

	Neighborhood Attribute	Psychological Distress		Depressive Symptoms	
		# Studies	# Studies with Significant Effect (Moderating Effect)	# Studies	# Studies with Significant Effect (Moderating Effect)
Residential	Average building height	--	--	1	1
	Dwelling level configuration	1	(1)	--	--
	% Residential area	--	--	1	1
	Residential development density	--	--	--	--
	Residential density	--	--	3	0
	Variation of building height	--	--	1	1
Infrastructure	Accessibility of streets	1	(1)	--	--
	Distance to public transportation	1	0	--	--
	Internal connectivity	--	--	1	0
	Land-use availability	--	--	1	(1)
	Land-use configuration	1	(1)	1	1
	Land-use mix	1	(1)	1	1
	Land-use diversity	--	--	1	0
	Major street	--	--	1	(1)
	Street connectivity	1	0	1	0
	Street intersection density	--	---	2	0
	Walkability	1	0	2	(1)
Sociodemographic	Cumulative exposure to deprivation	--	--	1	--
	Neighborhood deprivation	1	(1)	3	(1)
	Neighborhood poverty	--	--	2	1
	Neighborhood socioeconomic status	--	--	1	0
	Residential income level	--	--	1	--
	Residential density (population)	1	0	--	--
	Residential stability (population)	--	--	1	1
	Vehicle burden	--	--	1	(1)
Public services	Crime	--	--	1	1
	Distance to nearest public open space	--	--	1	0
	Size of nearest public open space	--	--	1	0
	Total number of public open spaces	1	0	1	0
	Total size of public open spaces	1	0	1	0
Environmental	Access to green qualities	2	(2)	--	--
	Blue space visibility	1	1	--	--
	Green exposure (NDVI)	2	1	4	1 (1)
	Green space visibility	1	0	--	--
	Green parkland ratio	--	--	1	(1)
	Nature diversity	--	--	1	0
	Nearest green space	--	--	1	1
	Nearest green or blue space	--	--	1	1
	Nearest green or agricultural space	--	--	1	1
	Nearest green, blue or agricultural space	--	--	1	1
	Slope variability	1	1	--	--
	% grass	1	(1)	--	--
	% green spaces	3	(2)	1	1
	% low-lying vegetation	1	0	--	--
	% tree canopy	1	1	1	1
Neighborhood resources	Density of businesses	--	--	1	0
	Geographical accessibility score	1	0	--	--
	Neighborhood resources	--	--	2	(2)
	Proximity to nearest PHCC	--	--	1	1
	Social engagement destinations	--	--	1	(1)

Note. NDVI, normalized difference vegetation index; PHCC, primary healthcare clinics.

## Data Availability

The data presented in this study are available on request from the corresponding author.

## Supplementary Materials

The following are available online at https://www.mdpi.com/article/10.3390/ijerph18168597/s1. Table S1. Associations of neighborhood attributes with psychological distress or depressive symptoms.

## Appendix A. Search Strategy

Ovid Medline; PsycInfo; Embase

1. exp Geographic Information Systems/
2. (geographic* information system* or GIS or geospatial or spatial analysis).mp.
3. 1 or 2
4. exp Mental Health/
5. exp Mental Disorders/
6. exp Mental Health Services/
7. exp Hospitals, Psychiatric/
8. (mental health or depress* or distress “psychological distress”).mp.
9. 4 or 5 or 6 or 7 or 8
10. 3 and 9
11. Limit 10 to (all journals and english language)

Scopus

1. (TITLE-ABS-KEY (“GIS” OR “geographic Information system” OR “geographical information system” OR “geographic information systems” OR geospatial OR “spatial analysis”) AND TITLE-ABS-KEY (mental AND health AND OR depress* OR distress OR “psychological distress”)) AND (LIMIT-TO (LANGUAGE, “English”))
2. Additional limit article

CINAHL

1. AB (“geographic information system” OR gis OR “geographical information system” OR (“geographic information systems” OR “geographical information systems” OR geospatial OR “spatial analysis”) AND AB (mental health OR depress* OR “psychological distress”)
2. Limit English and limit research article

Compedex; GEOBASE

1. ((((GIS OR “geographic information system” OR “geographical information system” OR “geographic information systems” OR “geographical information systems” OR geospatial OR “spatial analysis”) WN ALL) AND ((“mental health” OR depress* OR “psychological distress”) WN ALL))) AND ({english} WN LA))
2. Limit journal article
