# Supplementary material for: Use of Geographic Information Systems to Explore Associations between Neighborhood Attributes and Mental Health Outcomes in Adults: A Systematic Review
Source: Int J Environ Res Public Health. 2021 Aug 14;18(16):8597. doi: 10.3390/ijerph18168597 (PMC8393279; doi:10.3390/ijerph18168597)
Supplement: Supplementary file 1 [file ijerph-18-08597-s001.zip › ijerph-1320655-supple.pdf]

Supplementary Table S1. Associations of neighborhood attributes with psychological distress or depressive symptoms.

Author, Date	Outcome	Outcome Measure	Neighborhood Attribute(s)	Summary of Results
Ambrey, 2016a	Psychological distress	Kessler-10	• Green space (ha) per capita	- No significant association
Ambrey, 2016b	Psychological distress	Kessler-10	• Green space (km^2)	- No significant association<br>- Green space significantly linked to lower levels of psychological distress in adults who engage in physical activity living in more populated neighborhoods ($\beta = -0.61$ (SE = 0.19))
Annerstedt et al., 2012	Poor mental health	GHQ-12 (yes vs. no)	• Access to green qualities (Wild, Space, Serene, Culture, and Lush)	- No significant relationship with development of psychological distress at follow-up<br>- Significant association with lower risk of poor mental health in female adults, if access to Serene qualities and being physically active (OR = 0.2, 95% CI (0.06, 0.9)), if access to Space qualities and physically active (OR = 0.3, 95% CI (0.1, 0.9))
van den Bosch et al., 2015	Poor mental health	GHQ-12 (yes vs. no)	• Gained access to green qualities (Wild, Space, Serene, Culture, and Lush)	- No significant association of moving to an area with an increased number of nature qualities at baseline and follow-up<br>- Significant association between gained access to the nature quality of Serene and improved mental health among female adults only (OR = 4.51, 95% CI (1.29, 5.83))
Astell-Burt et al., 2013	Psychological distress	Kessler-10 (yes vs. no)	• % Green spaces	- Significant association of living in areas with more green spaces with a lower risk of psychological distress in middle-aged and above adults (Q2 (41–60%) OR = 0.93, 95% CI (0.88, 0.98), Q3 (61–80%) OR = 0.90, 95% CI (0.83, 0.97), Q4 (81%) OR = 0.91, 95% CI (0.84, 1.00) vs. Reference group (0–20%))
Astell-Burt et al., 2019	Psychological distress	Kessler-10 (yes vs. no)	• % Low-lying vegetation<br>• % Total green spaces<br>• % Grass<br>• % Tree canopy	- No significant association<br>- Inconsistent results with respect to total green space<br>- Inconsistent results with respect to grass<br>- Significant association of exposure to more tree canopy with lower prevalence (ranged from OR = 0.61 to OR = 0.81) and incidence (ranged from OR = 0.79 to OR = 0.92) of psychological distress in middle-aged and above adults
Berke et al., 2007	Depressive symptoms	CES-D 20 (yes vs. no)	• The walkability score	- Significant decrease in risk of depressive symptoms with living in more-walkable neighborhoods for male older adults only OR = 0.31 (95% CI

Author, Date	Outcome	Outcome Measure	Neighborhood Attribute(s)	Summary of Results
				(0.12, 0.81)) at 100 m buffer; OR = 0.32 (95% CI (0.13, 0.80)) at 500 m buffer; and OR = 0.33 (95% CI (0.14, 0.82)) at 1000 m buffer
Beyer et al., 2014	Depressive symptoms	DASS 42 score	• Level of greenness<br>• NDVI<br>• % Tree canopy coverage	- Significant association of living in areas with 25% higher levels of neighborhood green spaces with lower levels of depressive symptoms for adults (25% more green space β = −1.38 (SE = 0.40), 25% higher NDVI β = −1.37 (SE = 0.46), 25% more tree canopy, β = −1.01 (SE = 0.30))
Cromley et al., 2012	Depressive symptoms	CES-D 10 score	• Poverty<br>• Residential stability<br>• Crime	- Significant association of living in areas having higher poverty levels with higher levels of depressive symptoms for middle-aged and above adults in 80% of the census tracts<br>- Significant association of living in areas having higher residential stability with lower levels of depressive symptoms for middle-aged and above adults in the northern part of the New Jersey state<br>- Significant association of living in areas having higher crime rate per 1000 with higher levels of depressive symptoms for middle-aged and older adults in 60% of the census tracts
DeGuzman et al., 2013	Psychological distress	BSI-18 score	• Residential density<br>• Distance to public transportation	- No significant association<br>- No significant association
Francis et al., 2012	Psychological distress	Kessler-6 (yes vs. no)	• Total number of POS<br>• Total size of POS	- No significant association
Gariepy et al., 2015a	Depressive symptoms	PHQ-9 (yes vs. no)	• Neighborhood resources<br>• Greenness<br>• Neighborhood deprivation	- Significant associations between the number of physical activity facilities (AHR = 0.71, 95% CI (0.55, 0.91)) and cultural services (AHR = 0.75, 95% CI (0.57, 0.99)) and lower risk of depression in diabetic adults<br>- No significant association<br>- Significant moderating effect of being older (AHR = 1.01. p-value = 0.01) and retired (AHR = 1.28, p-value = 0.04) on deprivation increasing levels of depressive symptoms in diabetic adults

Author, Date	Outcome	Outcome Measure	Neighborhood Attribute(s)	Summary of Results
Gariepy et al., 2015b	Depressive symptoms	CIDI-SFMD (yes vs. no)	• Presence of any park, healthcare service, healthy food store, fast food restaurant, cultural service	- Significant association with presence of any neighborhood service (parks, healthy food stores, fast food restaurants, health service) except for cultural services, with a trajectory shift towards a low probability of having depressive symptoms in adults<br>- Significant association of presence of any park with a trajectory shift towards a lower and a moderate probability of having depressive symptoms in adults
Ho et al., 2017	Depressive symptoms	GDS-15 (yes vs. no)	• Variation of building height<br>• % Residential area<br>• Average building height<br>• % Vegetation (NDVI)	- 1% increase in the variation of building height had 3% increase in geriatric depression risk (OR = 1.03, 95% CI (1.01, 1.04))<br>- 1% increase in percentage of residential areas had 1% increase in geriatric depression risk (OR = 1.01, 95% CI (1.00, 1.02))<br>- 1% increase in average building height had lower geriatric depression risk (OR = 0.98, 95% CI (0.96, 0.99))<br>- No significant association with % vegetation
Ivey et al., 2015	Depressive symptoms	CES-D 10 (yes vs. no)	• Density of businesses<br>• Neighborhood SES	- No significant association<br>- Significant association of having fewer businesses within 400 m of home with decreased odds of having mild to moderate levels of depressive symptoms in the unadjusted model, not significant in adjusted model<br>- No significant association<br>- Significant association of living in a lower SES neighborhood with increased odds of depressive symptoms in the unadjusted model, not significant in adjusted model
Koohsari et al., 2018	Depressive symptoms	CES-D 10 (yes vs. no)	• Distance to the nearest POS<br>• Size of the nearest POS<br>• Total number of POS<br>• Total size of POS	- No significant association (<400 m)<br>- No significant association (>1.5 ha)<br>- No significant association<br>- No significant association

Author, Date	Outcome	Outcome Measure	Neighborhood Attribute(s)	Summary of Results
Mayne et al., 2018	Psychological distress	Kessler-10 (yes vs. no)	• Walkability index	- No significant association
Moore et al., 2016	Depressive symptoms	CES-D 20 score	• Social engagement destinations	- Significantly lower levels of depressive symptoms with the greater social engagement destination density in middle-aged females at baseline but not in males (AMD = −0.42, 95% CI (−0.82, −0.03))<br>- No significant changes over 10 years
			• %Green space	- Not significant within 300 m, 500 m, and 1000 m buffers at baseline<br>- No significant changes over 10 years within 300 m, 500 m, and 1000 m buffers
Noordzij et al., 2020	Depressive symptoms	MHI-5 scores	• Distance to nearest green; green or blue; green or agricultural; green, blue or agricultural space	- Significant at baseline (β = −0.494; β = −0.584; β = −0.445; β = −0.547)<br>- No significant changes over 10 years
Nutsford et al., 2016	Psychological distress	Kessler-10 score	• Green space visibility	- No significant association (≤300 m, 300 m–3 km, 3–6 km, 6–15 km)
			• Blue space visibility	- Significant association of the higher visibility of blue space >3 km with lower psychological distress in adults (β = −0.28, 95% CI (−0.41, −0.15))
Rantakokko et al., 2018	Depressive symptoms	CES-D 20 score	• Nature diversity	- No significant association
Saarloos et al., 2011	Depressive symptoms	GDS-15 (yes vs. no)	• Land-use mix	- Significantly higher levels of depressive symptoms in male older adults living in areas with more diverse land-use (T1 (low) OR = 1, T2 (middle) OR = 1.54, 95% CI (1.10, 2.16), OR = 1.52 (high), 95% CI (1.08, 2.14))
			• Land-use availability (retail)	- Significantly higher levels of depressive symptoms in male older adults living in areas with retail than in areas without retail (OR = 1.46, 95% CI (1.11, 1.90))
			• Land-use availability<br>• (other retail)	- No significant association

Author, Date	Outcome	Outcome Measure	Neighborhood Attribute(s)	Summary of Results
			• Land-use availability (offices/businesses)	- No significant association
			• Land-use availability<br>• (health/<br>• well-being/community services)	- No significant association
			• Land-use availability (entertainment/<br>• recreation/culture)	- No significant association
			• Walkability	- No significant association
			• Street connectivity	- No significant association
			• Residential density	- No significant association
Sakar et al., 2013	Psychological distress	GHQ-30 (yes vs. no)	• Dwelling level variables<br>- Dwelling type (semi-detached vs. detached)<br>- Dwelling type (terraced vs. detached)<br>- Dwelling type (flat vs. detached)	- No significant associations except for dwelling type (terraced vs. detached)<br>- Significant association of living in terraced houses with lower odds of psychological distress (OR = 0.48, 95% (−1.51, 0.02)) in male older adults
Sakar et al., 2013	Psychological distress	GHQ-30 (yes vs. no)	• Land-use configuration	- Significant association of land-use mix with lower odds of psychological distress (OR = 0.63, 95% (−1.10, 0.18)) in male older adults

Author, Date	Outcome	Outcome Measure	Neighborhood Attribute(s)	Summary of Results
			- Land-use mix<br>- Density of bus stops<br>- Density of businesses and offices<br>- Density of retail<br>- Density of community services<br>- Density of recreation and leisure facilities	- Significant association of density of bus stops with higher odds of psychological distress (OR = 1.04, 95% CI (−0.02, 0.10)) in male older adults<br>- Significant association of density of businesses and offices with higher odds of psychological distress (OR = 1.02, 95% CI (−0.01, 0.04)) in male older adults<br>- No significant association with density of retail, community services, and recreation and leisure facilities
			• Accessibility of streets<br>- Street movement potential R1200 m<br>- Street movement potential R3000 m<br>- Street movement potential RN m<br>- Connectivity	- No significant associations except with street movement potential R 1200 m<br>- Significant association of local-level street (<1200 m) accessibility with lower odds of psychological distress (OR = 0.54, 95% CI (−1.28, 0.03)) in male older adults
			• Green exposure (NDVI)	- No significant association
			• Slope variability	- Significant association of slope variability with higher odds of psychological distress (OR = 1.38, 95% CI (−0.07, 0.71)) in male older adults
			• Neighborhood deprivation<br>- Employment deprivation	- No significant association with income, health, education, and housing deprivation

Author, Date	Outcome	Outcome Measure	Neighborhood Attribute(s)	Summary of Results
			- Health deprivation<br>- Education deprivation<br>- Housing deprivation<br>- Income deprivation<br>- Physical environment	- Significant association of employment deprivation with lower odds of psychological distress (OR = 0.96, 95% CI (−0.08, 0.00)) in male older adults<br>- Significant association of physical environment deprivation with higher odds of psychological distress (OR = 1.02, 95% CI (0.00, 0.04)) in male older adults
Schootman et al., 2007	Depressive symptoms	CES-D 11 (yes vs. no)	• Block group deprivation indices<br>• Census tract deprivation indices	- No significant association<br>- No significant association
Song et al., 2007	Depressive symptoms	SCL-90-R score	• Vehicle burden<br>• Neighborhood poverty<br>• Connectivity<br>• Major streets<br>• Land-use diversity<br>• Green parkland ratio<br>• Residential density	- No significant main effects of any neighborhood attributes<br>- Significant cross-level interactions between perceived traffic stress and vehicle burden, major streets and parks (associations between traffic stress and depressive symptoms were greater for residents living in neighborhoods with a greater vehicular burden (γ = 0.002, SE = 0.001) and with major streets (γ = 0.003, SE = 0.001), and weaker for those living in neighborhoods with a higher green parkland ratio (γ = −0.006, SE = 0.011))
Su et al., 2019	Psychological distress	GHQ-12 (yes vs. no)	• Green exposure (NDVI)	- Significant association of higher vegetation index with lower risks of psychological distress in adults across 50, 100, 250, and 500 m buffers

Author, Date	Outcome	Outcome Measure	Neighborhood Attribute(s)	Summary of Results
Thomas et al., 2007	Common mental health symptoms	PHQ-12 score	• Geographical accessibility score	- No significant association<br>- Geographical accessibility of leisure and entertainment facilities was most strongly associated, but not significant
Tomita et al., 2017a	Depressive symptoms	CES-D 20 (yes vs. no)	• Green exposure (NDVI)	- Significant association of living in areas having higher green exposure with lower risks of depressive symptoms in middle-income adults compared with low-income adults (aOR = 0.98, 95% CI (0.97, 0.99))
Tomita et al., 2017b	Depressive symptoms	CES-D 20 (yes vs. no)	• Proximity to the nearest PHCC	- Significant association of living away from a PHCC (15 km≥) with higher risks of depressive symptoms in adults (T1 (15 km≥) OR = 1, T2 (6–14.9 km) OR = 0.61, 95% CI (0.44–0.86), T3 (<6 km) OR = 0.61, 95% CI (0.42, 0.87))
Traoré et al., 2020	Depressive symptoms	MINI (yes vs. no)	• Income level<br>• Cumulative exposure deprivation	- Significant associations of residing in low (OR = 2.36, 95% CI (1.57, 3.54)) and average-income neighborhoods (OR = 2.16, 95% CI (1.43, 3.25)) compared to residing in high-income neighborhoods<br>- Significant associations for individuals who frequented neighborhoods of different types (OR = 2.04, 95% CI (1.32, 3.29)) and who frequented only poor neighborhoods (OR = 1.72, 95% CI (1.16, 2.55)) compared to individuals who frequented wealthy neighborhoods only
van den Bosch et al., 2015	Poor mental health	GHQ-12 (yes vs. no)	• Gained access to green qualities (Wild, Space, Serene, Culture, and Lush)	- No significant association of moving to an area with increased access to a number of nature qualities at baseline and follow-up<br>- Significant association between gained access to the nature quality of Serene and improved mental health in female adults only (OR = 4.51, 95% CI (1.29, 5.83))
Zhang et al., 2018	Depressive symptoms	GDS-4 score	• Residential density<br>• Street intersection density<br>• Prevalence/number of neighborhood resources	- No significant associations with (density of: gross residential, street intersection, civic and institutional, retail, entertainment, recreational, food-related destination, public transport; Prevalence of: non-food retail and services, food-related shops, eating outlets, public transport stops, health clinics/services; connectivity; recreational destination diversity; number of parks; pedestrian infrastructure)<br>- Significant moderating effect of living alone in areas having poorer access to civic/institutional destinations, retail, food/eating outlets, public transport stops and health clinics/services increasing levels of depressive symptoms in older adults (all significant at *p*-value < 0.01 level)<br>- No significant associations

Author, Date	Outcome	Outcome Measure	Neighborhood Attribute(s)		Summary of Results
Zhang et al., 2019	Depressive symptoms	GDS-4 score	• Residential density<br>• Street intersection density<br>• Prevalence/number of neighborhood resources	-	Frequency of walking for transport was negatively related to the likelihood of experiencing depressive symptoms and a partial mediator of the interaction effect of living arrangement and destinations on depressive symptoms in older adults

Note. Explanations of acronyms are in alphabetical order: AHR, adjusted hazards ratio; AMD, adjusted mean differences; aOR, adjusted odds ratio; BSI-18, Brief Symptom Inventory-18; CES-D, Center for Epidemiologic Studies short Depression Scale; CI, confidence interval; CIDI-SFMD, Composite Diagnostic Interview Short-Form for Major Depression; DASS, Depression Anxiety and Stress Scales; GDS, Geriatric Depression Scale; GHQ, General Health Questionnaire; GIS, Geographic Information Systems; MHI, mental health inventory; MINI, Mini-International neuropsychiatric interview; NDVI, normalized difference vegetation index; OR, odds ratio; PHQ, Patient Health Questionnaire; PHCC, primary healthcare clinics; POS, public open spaces; Q, quartile; SE, standard deviation; SES, socioeconomic status; SCL-90-R, Revised Symptom Checklist 90; T, tertile; WNWT, within-neighborhood walking for transport.
